# Auswirkungen auf die Arzneimitteltherapiesicherheit und Adhärenz in der Dermato‐Onkologie: Das AMBORA‐Therapiebegleitungskonzept für orale Antitumortherapeutika

**DOI:** 10.1111/ddg.15809_g

**Published:** 2025-10-23

**Authors:** Lisa Cuba, Frank Dörje, Rafaela Kramer, Pauline Dürr, Michael Erdmann, Martin F. Fromm, Carola Berking, Katja Gessner

**Affiliations:** ^1^ Apotheke des Universitätsklinikums Erlangen und Friedrich‐Alexander‐Universität Erlangen‐Nürnberg Erlangen Deutschland; ^2^ Institut für Experimentelle und Klinische Pharmakologie und Toxikologie Friedrich‐Alexander‐Universität Erlangen‐Nürnberg Erlangen Deutschland; ^3^ Comprehensive Cancer Center (CCC) Erlangen‐EMN CCC WERA Universitätsklinikum Erlangen und Friedrich‐Alexander‐Universität Erlangen‐Nürnberg Erlangen Deutschland; ^4^ Bayerisches Zentrum für Krebsforschung (Bavarian Center for Cancer Research, BZKF) Erlangen Deutschland; ^5^ Apotheke der Klinik Floridsdorf Wiener Gesundheitsverbund Wien Österreich (aktuelle Anschrift); ^6^ FAU NeW – Forschungszentrum Neue Wirkstoffe Friedrich‐Alexander‐Universität Erlangen‐Nürnberg Erlangen Deutschland; ^7^ Hautklinik Universitätsklinikum Erlangen und Friedrich‐Alexander‐Universität Erlangen‐Nürnberg Erlangen Deutschland

**Keywords:** Basalzellkarzinom, Melanom, Pharmakologie, Proteinkinase, T‐Zell‐Lymphom, Basal cell carcinoma, melanoma, pharmacology, protein kinase, T‐cell lymphoma

## Abstract

**Hintergrund:**

Dermatologische orale Antitumortherapeutika (OAT) sind häufig interaktionsanfällig und werden in komplexen Therapieschemata eingesetzt. Das klinisch‐pharmakologische/pharmazeutische Therapiebegleitungskonzept der randomisierten AMBORA‐Studie verbesserte die Arzneimitteltherapiesicherheit bei verschiedensten OAT signifikant, dermato‐onkologische Patienten waren jedoch nicht eingeschlossen. Das Konzept wurde im Anschluss in die klinische Routine implementiert und die Dermato‐Onkologie eingeschlossen. Ziel dieser Untersuchung war die Analyse von Medikationsfehlern sowie der Adhärenz bei Patienten mit dermatologischen OAT.

**Patienten und Methodik:**

Medikationsfehler wurden unter anderem nach Ursache charakterisiert (PCNE V9.1). Die Adhärenz wurde mit dem „Medication‐Event‐Monitoring‐System“ MEMS^®^ Button und dem MARS‐D Fragebogen erfasst. Primäre Endpunkte waren der Anteil an gelösten OAT‐bezogenen Medikationsfehlern und die *Dosing Adherence (DA)*, (Tage mit korrekter OAT‐Einnahme) über 12 Wochen.

**Ergebnisse:**

Bei 92 Patienten (81,5% mit Melanom) wurden im Mittel 1,6 Medikationsfehler pro Patient festgestellt. Davon betrafen 61,6% die OAT, von denen 89,2% behoben wurden. Von 52 Patienten, die am zusätzlichen Adhärenzmonitoring teilnahmen, waren 48 evaluierbar und erreichten eine mediane *DA* von 95,0% sowie einen MARS‐D‐Score von 25/25. Die *DA* war bei OAT mit 1 x täglicher Einnahme höher als bei 2 x täglicher (p = 0,0127).

**Schlussfolgerungen:**

Das interprofessionelle AMBORA‐Therapiebegleitungskonzept in der Dermato‐Onkologie war mit einem großen Anteil gelöster Medikationsfehler und hoher Adhärenz assoziiert. Evidenzbasiertes Medikationsmanagement und Patientenberatungen und Patientenberatung durch klinische Pharmakologen/Pharmazeuten optimieren die Arzneimitteltherapiesicherheit in der dermato‐onkologischen Versorgung.

## EINLEITUNG

Orale Antitumortherapeutika (OAT) werden zunehmend in verschiedenen Bereichen eingesetzt, darunter auch in der Dermato‐Onkologie.^1^ Mit der Einführung von BRAF‐ und MEK‐Inhibitoren hat die Behandlung des Melanoms seit 2012 deutlich weiterentwickelt.^2^ Aktuell stehen drei verschiedene BRAF/MEK‐Kombinationsschemata zur Verfügung, die fester Bestandteil der modernen Melanomtherapie sind.^3^ Darüber hinaus sind die Hedgehog‐Inhibitoren Sonidegib und Vismodegib zur Behandlung des Basalzellkarzinoms verfügbar und das Retinoid Bexaroten stellt eine orale Therapieoption beim kutanen T‐Zell‐Lymphom dar.^3^

Orale Antitumortherapeutika bieten verschiedene Vorteile für Patienten und Behandlungsteams, zum Beispiel eine bequemere Anwendung im Vergleich zu intravenösen Therapien.^4^ Allerdings kann der Therapieerfolg durch unsachgemäße Anwendung, unzureichende Adhärenz oder Interaktionen mit Arznei‐ oder Nahrungs(ergänzungs)mitteln gefährdet werden.^1^ Die Adhärenz reicht von 14% bis 100% und ist zum Beispiel abhängig von der Patientenkohorte, den Dosierungsschemata oder den Bewertungsmethoden.^5^, ^6^ So war beispielsweise die Adhärenz bei Imatinib der einzige unabhängige Prädiktor für den Therapieerfolg bei chronisch myeloischer Leukämie: Bei Patienten mit einer Adhärenz von < 80% wurde kein molekulares Ansprechen beobachtet.^7^ Dermatologische OAT sind bezüglich der Adhärenz besonders interessant, da sie häufig in komplexen Therapieschemata eingenommen werden (zum Beispiel Abstand zur Nahrungsaufnahme bei Dabrafenib/Trametinib oder komplizierte Therapieschemata bei Vemurafenib [2 x täglich, kontinuierlich] und Cobimetinib [1 x täglich, zyklisch]).^8^

Die randomisierte, multizentrische AMBORA‐Studie (Arzneimitteltherapiesicherheit bei der Behandlung mit neuen oralen Antitumor‐Wirkstoffen, 2017–2020) untersuchte ein interprofessionelles Therapiebegleitungskonzept zur Optimierung der Arzneimitteltherapiesicherheit bei der Behandlung mit verschiedensten OAT – mit Ausnahme von dermato‐onkologischen Patienten.^9^, ^10^ Die Patienten wurden über 12 Wochen nach Beginn einer oralen Antitumortherapie zu vier Zeitpunkten von klinischen Pharmakologen/Pharmazeuten zusätzlich zu den Routineterminen beraten. Das Therapiebegleitungskonzept umfasste vier Bausteine: *(1)* umfassende Medikationsanalysen, bei denen die gesamte Medikation evaluiert wurde, *(2)* strukturierte Beratungsgespräche zu den OAT (zum Beispiel zur korrekten Einnahme), *(3)* Management von Nebenwirkungen und *(4)* Optimierung der Adhärenz durch das Angebot verschiedener Techniken (zum Beispiel Apps, Tagebücher).^9^ Medikationsfehler (zum Beispiel Interaktionen zwischen Arznei‐ und Nahrungs[ergänzungs]mitteln) wurden in der AMBORA‐Studie häufig festgestellt (1,7 pro Patient innerhalb von 12 Wochen nach Beginn der OAT).^10^ Die subjektive Adhärenz der oralen Antitumortherapie war in der AMBORA‐Studie hoch.^9^

Das AMBORA‐Therapiebegleitungskonzept wurde im Rahmen des von der Deutschen Krebshilfe geförderten AMBORA‐Kompetenz‐ und Beratungszentrums (AMBORA‐Zentrum) an unserem universitären *Comprehensive Cancer Center* in die klinische Routineversorgung implementiert.^11^ Im Zuge dessen wurden erstmals Patienten aus der Dermato‐Onkologie eingeschlossen. Ziel dieser Untersuchung war es, bei dermato‐onkologischen Patienten, die mit OAT behandelt wurden, *(1)* Medikationsfehler zu identifizieren, zu charakterisieren und zu beheben, *(2)* die objektive und subjektive Adhärenz zu beschreiben und *(3)* gezielte Empfehlungen zur Optimierung der Arzneimitteltherapiesicherheit bei OAT in der Dermato‐Onkologie abzuleiten.

## PATIENTEN UND METHODIK

### Studiendesign und Patienten

Diese prospektive Untersuchung wurde im AMBORA‐Zentrum^11^, ^12^ innerhalb des universitären *Comprehensive Cancer Center Erlangen‐EMN* durchgeführt. Sie wurde von der Ethikkommission der Friedrich‐Alexander‐Universität Erlangen‐Nürnberg genehmigt und beim Deutschen Register für Klinische Studien registriert (DRKS00026272).

Zweiundneunzig Patienten aus der Dermato‐Onkologie, die OAT einnahmen, wurden zwischen September 2021 und Oktober 2023 von klinischen Pharmakologen/Pharmazeuten des AMBORA‐Zentrums nach erfolgter Einverständniserklärung beraten. Die vier bereits beschriebenen Bausteine wurden im intensivierten AMBORA‐Therapiebegleitungskonzept adressiert (umfassende Medikationsanalysen, strukturierte OAT‐Beratung, Management von Nebenwirkungen und Optimierung der Adhärenz).^9^ Die Beratung basierte auf den in der randomisierten AMBORA‐Studie^9^ etablierten Arbeitsanweisungen (SOP) und umfasste unter anderem systematisch entwickeltes Informationsmaterial für Patienten (zum Beispiel Merkblätter zu Antitumor‐Medikamenten oder häufigen Nebenwirkungen). Alle Patienten, die zu Beginn der OAT (≤ 1 Woche Einnahme) zum ersten Mal beraten wurden, konnten fest definierte Folgegespräche über 12 Wochen in Anspruch nehmen. Patienten, die während einer bereits laufenden OAT‐Behandlung zum ersten Mal beraten wurden, erhielten Folgegespräche nur bei Bedarf (zum Beispiel im Falle von Nebenwirkungen).

**ABBILDUNG 1:**
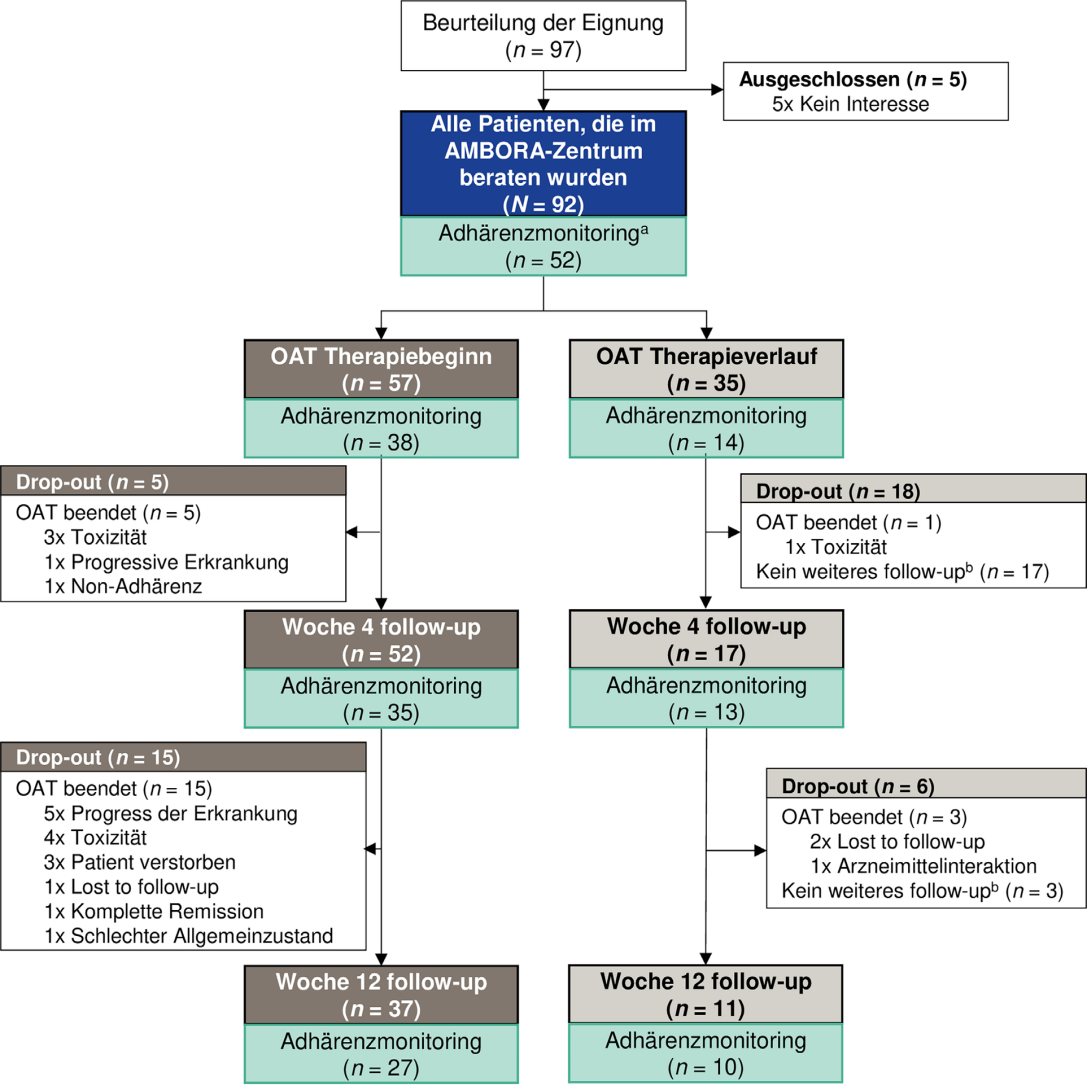
CONSORT‐Diagramm der Patienten, die im AMBORA‐Zentrum beraten und mit dermatologischen OAT behandelt wurden. Patienten stratifiziert nach Zeitpunkt der Erstberatung (Therapiebeginn oder Therapieverlauf) und Teilnahme am Adhärenzmonitoring. *Abk*.: OAT, orale Antitumortherapeutika. ^a^Vierzig Patienten nahmen nicht am Adhärenzmonitoring teil (33 x Teilnahme wurde nicht angeboten, 3 x kein Interesse, 2 x sprachliche Barriere und 2 x kognitive Einschränkungen). ^b^Folgegespräche wurden für Patienten, die im OAT Therapieverlauf erstmals beraten wurden, nur bei Bedarf angeboten (zum Beispiel aufgrund von Nebenwirkungen).

Zwischen Oktober 2022 und Oktober 2023 führten wir ein zusätzliches Adhärenzmonitoring durch (Abbildung 1). Für diese 52 Patienten wurden Folgegespräche nach 4 und 12 Wochen durchgeführt – unabhängig vom Zeitpunkt der ersten Beratung (Therapiebeginn oder Therapieverlauf).

**(1) Bewertung von Medikationsfehlern**

Während aller Beratungsgespräche wurden umfassende Medikationsanalysen durchgeführt, die auf den in der AMBORA‐Studie etablierten SOP basierten.^9^, ^10^ Die Gesamtmedikation (einschließlich OAT, weiterer verordneter Begleitmedikation sowie rezeptfreier [over‐the‐counter, OTC] Arzneimittel und Nahrungs[ergänzungs]mittel) wurde sorgfältig überprüft. Für weitere Informationen verweisen wir auf das Online‐Supplement.

Medikationsfehler wurden gemäß dem National Council for Medication Error Reporting and Prevention (NCC‐MERP) definiert als „jedes vermeidbare Ereignis, das zu einer unangemessenen Medikamentenanwendung oder zu einer Schädigung des Patienten führen kann“.^13^ Zu Medikationsfehlern zählen beispielsweise eine medikamentöse Therapie ohne Indikation, unbehandelte Indikationen, Dosierungsfehler, Interaktionen zwischen Arzneimitteln oder Nahrungs(ergänzungs)mitteln oder Anwendungsfehler durch die Patienten. Medikationsfehler wurden mithilfe validierter Instrumente klassifiziert: Pharmaceutical Care Network Europe (PCNE) V9.1^14^ wurde zur Charakterisierung von Ursache und Status verwendet, der Schweregrad mit dem NCC‐MERP‐Index^15^ bewertet. Bei allen Fehlern wurde die betroffene Medikation erfasst (Gesamtmedikation bestehend aus OAT und Begleitmedikation). Der primäre Endpunkt war die Reduktion der Anzahl an OAT‐bezogenen Medikationsfehlern über die Zeit. Medikationsfehler galten als gelöst, wenn sie nach der Durchführung der vorgeschlagenen Intervention (zum Beispiel Absetzen/Ansetzen eines Arzneimittels, Dosisänderung oder Patientenschulung) nicht mehr bestanden.

**(2) Adhärenzmonitoring**

#### Objektive Medikamentenadhärenz

Die Adhärenz wurde mit Hilfe des MEMS^®^‐Buttons^16^ (AARDEX^®^ Group, Seraing, Belgien) über 12 Wochen erfasst. Die Patienten wurden angewiesen, 1 x pro OAT und Einnahmezeitpunkt einen separaten Button zu drücken, unabhängig von der Anzahl der eingenommenen Tabletten/Kapseln oder dem erforderlichen Zeitabstand zur Nahrungsaufnahme (siehe Online‐Supplement).

Die Adhärenzparameter wurden in Übereinstimmung mit der ABC‐Taxonomie für Medikamentenadhärenz spezifiziert.^17^ Als primärer Endpunkt wurde hier die Anzahl der Tage mit korrekter Anzahl von OAT‐Einnahmen (= MEMS^®^‐Knopfdruck) bezogen auf die beobachteten Tage definiert (*Dosing Adherence, DA*). In Übereinstimmung mit zuvor verwendeten und international etablierten Grenzwerten^7^, ^18^, ^19^ wurden Patienten als non‐adhärent eingestuft, wenn die Gesamt‐*DA* ≤ 80% lag. Fehlende Datenpunkte ohne dokumentierte Gründe seitens der Patienten oder der elektronischen Patientenakte (zum Beispiel ärztlich angeordnete Therapiepausen aufgrund von Nebenwirkungen) wurden als ausgelassene Dosen gezählt. So wurden zum Beispiel Therapiepausen aufgrund von Fieber nicht als Non‐Adhärenz gewertet, da die Patienten ausdrücklich zu diesem empfohlenen Nebenwirkungsmanagement geschult wurden.

#### Subjektive Medikamentenadhärenz

Die patientenberichtete Adhärenz wurde mit dem validierten deutschen MARS‐D‐Fragebogen^20^ nach 4 und 12 Wochen erfasst. Der MARS‐D‐Fragebogen umfasst fünf Fragen zur Medikamenteneinnahme, die auf einer Skala von 1 bis 5 bewertet werden, was zu einem Gesamtscore von 5 bis 25 führt (25 = vollständig adhärent).^20^

### Datenanalyse

Die Daten wurden mit Microsoft Access^®^, Excel^®^ und der MEMS^®^ Adherence‐Software (AARDEX^®^ Group) deskriptiv ausgewertet. Die statistischen Tests basierten auf dem Intention‐to‐treat‐Prinzip und wurden mit 95%‐Konfidenzintervallen in GraphPad Prism^®^ durchgeführt. Kategorische Parameter (zum Beispiel Merkmale von Medikationsfehlern) wurden mithilfe des zweiseitigen Chi^2^‐Tests oder des exakten Fisher‐Tests analysiert. Kontinuierliche Variablen (zum Beispiel Adhärenzdaten) wurden mit dem parametrischen t‐Test, Mann‐Whitney‐Test oder Wilcoxon‐Rangsummentest ausgewertet.

## ERGEBNISSE

### Patientencharakteristika

Es wurden 92 dermato‐onkologische Patienten, die mit neun verschiedenen OAT behandelt wurden, im AMBORA‐Zentrum beraten (Tabelle 1). Die Mehrheit der Patienten (62,0%; 57/92) erhielt die Beratung zu Therapiebeginn der OAT (Abbildung 1). Bei 76,1% (70/92) aller Patienten wurde mindestens ein Folgegespräch durchgeführt. Die Charakteristika der Patienten, die zu Beginn der OAT oder während einer laufenden Therapie beraten wurden, waren vergleichbar (Tabelle S1, Online‐Supplement).

**TABELLE 1:** Charakteristika der Patienten, die im AMBORA‐Zentrum beraten und mit dermatologischen OAT behandelt wurden.

Patientencharakteristika	Anzahl an Patienten (%) N = 92
Alter, Jahre (Mittelwert)	61,1 [29–90]
Weibliches Geschlecht	52 (56,5)
ECOG 0–1	66 (71,7)
ECOG > 1	26 (28,3)
Berufstätig	22 (23,9)
Unterstützung im Alltag benötigt	17 (18,5)
Grapefruitkonsum	12 (13,0)
Einnahme von ≥ 1 OTC‐Arzneimitteln^a^	64 (69,6)
**Medikation pro Patient** (Median)	
Gesamtmedikation^b^	8 [1–22]
Orale Antitumortherapeutika^c^	2 [1–3]
Begleitmedikation	6 [0–20]
OTC‐Arzneimittel^a^	1 [0–10]
**Lebenssituation**	
Mit Partner/Familie	71 (77,2)
Alleinstehend	16 (17,4)
In Pflegeeinrichtung	4 (4,3)
NA	1 (1,1)
**Tumorart**	
Melanom	75 (81,5)
Kutanes T‐Zell‐Lymphom	11 (12,0)
Basalzellkarzinom	6 (6,5)
**Orale Antitumortherapeutika** ^d^	
Dabrafenib/Trametinib	49 (53,3)
Encorafenib/Binimetinib	18 (19,6)
Bexaroten	10 (10,9)
Temozolomid	4 (4,3)
Vemurafenib/Cobimetinib	3 (3,3)
Sonidegib	3 (3,3)
Vismodegib	3 (3,3)
Lenvatinib	1 (1,1)
Acitretin	1 (1,1)
**Behandlungscharakteristika**	
Zyklische Einnahme	8 (8,7)
Kurativ/adjuvant	23 (25,0)
Off‐Label‐Einsatz^e^	8 (8,7)

*Abk*.: ECOG, Eastern Cooperative Oncology Group; NA, nicht zutreffend; OAT, orale Antitumortherapeutika; OTC, *over‐the‐counter* (freiverkäuflich)

*Anmerkung*: Die Charakteristika gelten für den Beginn der Untersuchung (Zeitpunkt der ersten Beratung). Kategorische Variablen angegeben als Anzahl (%) der Patienten pro Gruppe, kontinuierliche Variablen als Mittelwert oder Median [Spannweite].

ns, nicht signifikant (t‐Test, Mann‐Whitney‐Test, Chi^2^‐Test oder Fishers’ Exact Test).

^a^ Umfasst OTC‐Arzneimittel und Nahrungs(ergänzungs)mittel.

^b^ Umfasst Arzneimittel aller Applikationsarten (zum Beispiel oral, parenteral oder topisch) und OTC‐Arzneimittel, sowie Nahrungs(ergänzungs)mittel.

^c^ Zwei Patientinnen mit Melanom, die mit Dabrafenib/Trametinib behandelt wurde, erhielten Exemestan bzw. Talazoparib gegen Brustkrebs. Ein Patient wurde mit Bexaroten und Methotrexat behandelt.

^d^ Umfasst nur OAT, die für dermato‐onkologische Indikationen verschrieben wurden.

^e^ Umfasst die palliative Therapie mit Temozolomid, Lenvatinib, Acitretin und die adjuvante Therapie mit Encorafenib/Binimetinib.

Das Adhärenzmonitoring wurde im definierten Zeitraum 59 Patienten angeboten und 52 (88,1%) stimmten der Teilnahme zu. Davon absolvierten 92,3% (48/52) und 71,5% (37/52) die Folgegespräche in Woche 4 bzw. Woche 12 (Abbildung 1). Es zeigten sich keine Unterschiede in den Charakteristika der Teilnehmer des Adhärenzmonitorings im Vergleich zu den übrigen Patienten (Tabelle S2, Online‐Supplement).

**(1) Medikationsfehler**

#### Anzahl an Medikationsfehlern

**ABBILDUNG 2:**
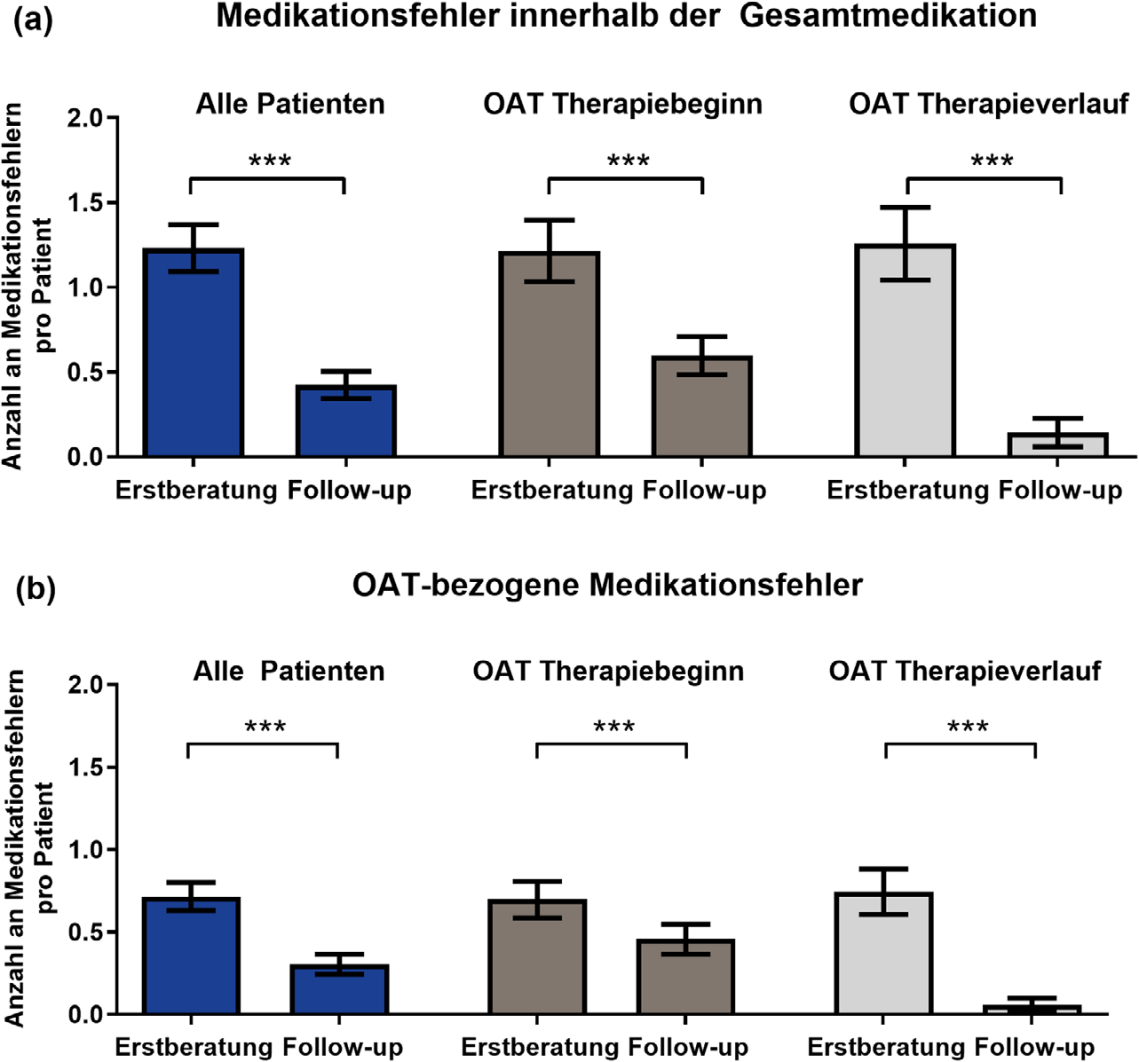
Anzahl an Medikationsfehlern pro Patient und die betroffene Medikation. Medikationsfehler stratifiziert nach Zeitpunkt der Erfassung (erstes Beratungsgespräch oder Folgegespräche) und dargestellt für: (a) Medikationsfehler innerhalb der Gesamtmedikation und (b) Medikationsfehler mit OAT‐Bezug. *Abk*.: OAT, orale Antitumortherapeutika. Daten angegeben als Mittelwert ± Standardfehler des Mittelwerts (SEM), ***p < 0,001 (Mann‐Whitney‐ oder Wilcoxon‐Rangsummentest).

Von allen Patienten hatten 78,3% (72/92) mindestens einen Medikationsfehler, wobei die meisten im ersten Beratungsgespräch auftraten (Abbildung 2). Insgesamt wurden 151 Fehler in der Gesamtmedikation (OAT und Begleitmedikation) detektiert (Mittelwert 1,6 pro Patient; Spannweite 0–11; vom ersten bis zum letzten Beratungsgespräch) (Abbildung 2a). Davon betrafen mehr Medikationsfehler die OAT im Vergleich zur Begleitmedikation (Mittelwert 1,0; Spannweite 0–11 vs. 0,6; 0–8; *p* < 0,0006) (Abbildung 2b). Über die Zeit wurden 87,4% (132/151) der Medikationsfehler in der Gesamtmedikation und 89,2% (83/93) der OAT‐bezogenen Fehler vollständig gelöst.

#### Charakteristika der OAT‐bezogenen Medikationsfehler

**ABBILDUNG 3:**
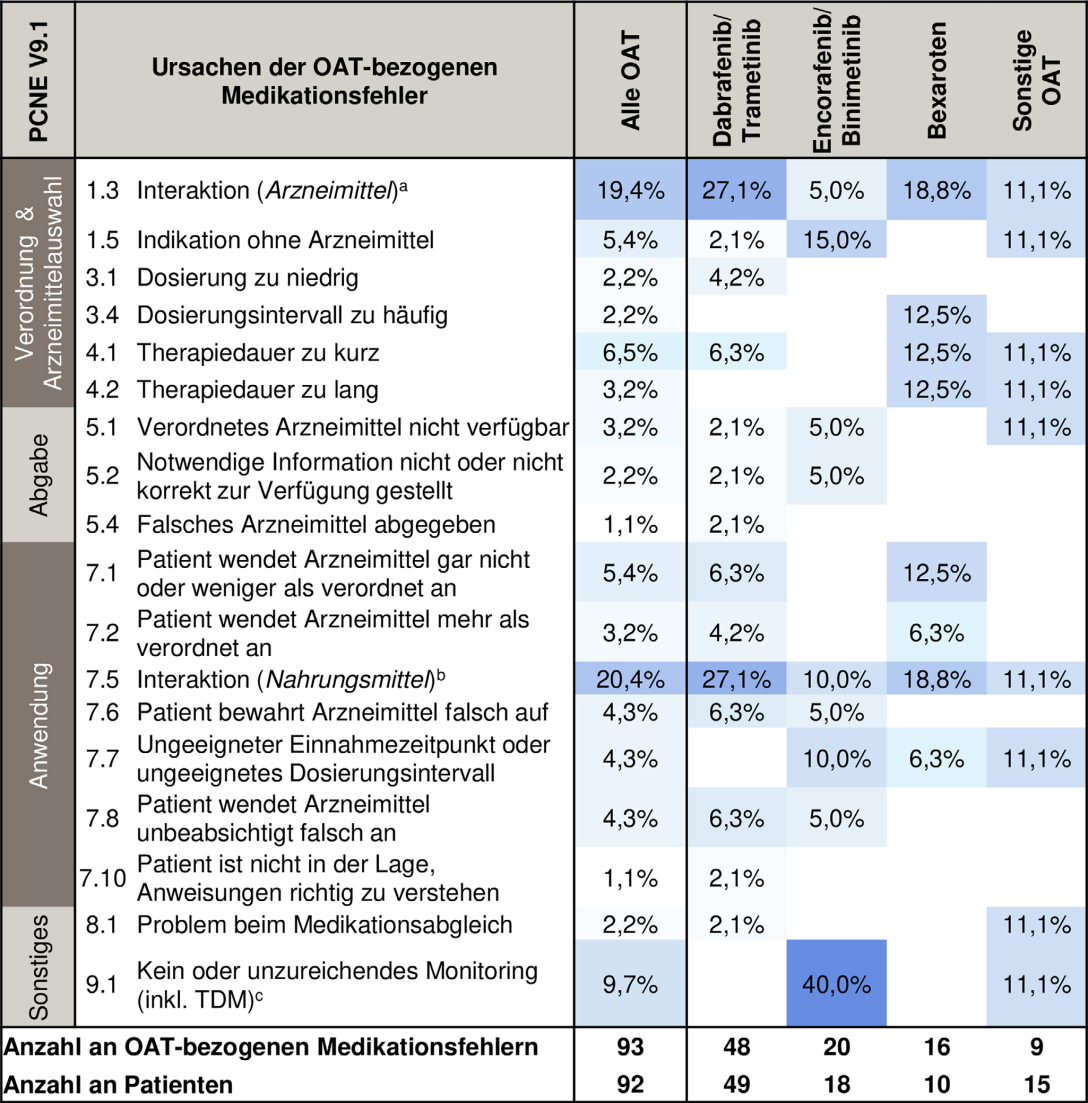
Heatmap der OAT‐bezogenen Medikationsfehler und deren Ursachen. Medikationsfehler stratifiziert nach den jeweiligen OAT‐Therapieschemata. *Abk*.: CYP, Cytochrom P450; OAT, orale Antitumortherapeutika; PCNE, Pharmaceutical Care Network Europe; TDM, Therapeutisches Drug Monitoring. Daten in Prozent (prozentualer Anteil der Medikationsfehler nach der jeweiligen Ursache [PCNE V9.1^14^] an der Gesamtanzahl aller OAT‐bezogenen Medikationsfehler pro Gruppe). ^a^Zum Beispiel Interaktion zwischen Dabrafenib (moderater CYP3A4 Induktor) und Sirolimus (CYP3A4 Substrat) bei einem nierentransplantierten Melanompatienten. Ein empfohlenes *T* *herapeutisches* *Drug Monitoring* zeigte Sirolimus‐Werte außerhalb des therapeutischen Bereichs. ^b^Umfasst OTC‐Arzneimittel und Nahrungs(ergänzungs)mittel (zum Beispiel gleichzeitige Einnahme von Bexaroten und Vitamin A oder Dabrafenib und Grapefruitsaft). ^c^Zum Beispiel fehlendes Elektrokardiogramm bei einem Patienten mit Hypokaliämie unter Amiodaron‐Therapie vor Wiederaufnahme von Encorafenib/Binimetinib. Feststellung einer schwerwiegenden QT‐Verlängerung nach erfolgter Intervention.

Interaktionen mit OAT‐Bezug (19,4%; 18/93) wurden häufig als Ursache für Medikationsfehler beobachtet (PCNE C1.3) (Abbildung 3). Ausgewählte Beispiele für OAT‐bezogene Medikationsfehler, stratifiziert nach allen PCNE‐Ursachen, sind in der Tabelle S3 (Online‐Supplement) aufgeführt. Bemerkenswerterweise hatten 43,0% (40/93) der Fehler mit OAT‐Bezug patientenbezogene Ursachen (PCNE C7.1–7.10, zum Beispiel Non‐Adhärenz, unangemessene Dosierungsintervalle) (Abbildung 3). Vorherrschend waren Interaktionen mit OTC‐Arzneimitteln, Nahrungs(ergänzungs)mitteln oder Grapefruit(‐produkten) (20,4%; 19/93). Das AMBORA‐Therapiebegleitungskonzept verhinderte, dass 33,3% (31/93) aller OAT‐bezogenen Fehler die Patienten erreichten (NCC‐MERP B). Insgesamt waren 6,5% (6/93) der OAT‐bezogenen Fehler mit Patientenschaden verbunden (NCC‐MERP ≥ E).

**(2) Adhärenzparameter**

Beim primären Endpunkt erreichten die Patienten im Median eine objektive *Dosing Adherence* (*DA*) von 95,0% (Spannweite 5,4–100,0) (Tabelle 2) über 12 Wochen. Die Patienten benutzten die MEMS^®^ Buttons 71 Tage lang (Median, 1–121). Die subjektive Adhärenz war hoch (medianer MARS‐D‐Score 25; 24–25). Beim Vergleich der *DA* und der subjektiven Adhärenz in den ersten 4 Wochen der Verwendung der MEMS^®^ Buttons zu den folgenden 8 Wochen zeigte sich kein Unterschied (Online‐Abbildung S1).

**TABELLE 2:** Adhärenzparameter aller evaluierbaren Patienten, die am Adhärenzmonitoring teilgenommen haben.

Adhärenzparameter	Median [Spannweite]
	Evaluierbare Patienten n = 48
**Objektive Adhärenz (MEMS^®^ Buttons)**	
*Dosing Adherence* (%)	
Gesamt	95,0 [5,4–100,0]
OAT 1 x täglich^a^	96,4 [10,7–100,0]
OAT 2 x täglich^b^	92,8 [0,0–100,0]
*Taking Adherence* (%)	
Gesamt	97,8 [7,1–103,3]
OAT 1 x täglich^a^	98,5 [10,7–112,5]
OAT 2 x täglich^b^	96,2 [3,6–100,0]
*Timing Adherence* (%)	
Gesamt	99,6 [75,0–100,0]
OAT 1 x täglich^a^	100,0 [66,7–100,0]
OAT 2 x täglich^b^	100,0 [90,8–100,0]
*Therapiebeginn* (Tage, Mittelwert)^c^	0,4 [0,0–5,0]
*Drug Holidays*	
Anzahl pro Patient	0,0 [0–10]
Anzahl an Patienten mit *≥ 1 Drug Holiday (*%)	19 (39,6)
Dauer (Tage, Mittelwert)	1,9 [0,0‐30,0]
*Persistenz*	
Anzahl an Patienten mit OAT‐Therapieabbrüchen (%)	3 (6,3)
**Zeit, die die MEMS** ^**®**^ **Buttons verwendet wurden (Tage, Mittelwert)**	
Gesamt	70,5 [1,0–121,0]
OAT 1 x täglich^a^	70,0 [1,0–‐121,0]
OAT 2 x täglich^b^	77,0 [1,0–121,0]
**Subjektive (patientenberichtete) Adhärenz**	
MARS‐D‐Gesamtscore	25 [24–25]
MARS‐D (%)	100,0 [95,0–100,0]

*Abk*.: MARS‐D, Medication Adherence Reporting Scale, validierte deutsche Übersetzung; MEMS, Medication Event Monitoring System; OAT, orale Antitumortherapeutika.

*Anmerkung*: Kategorische Variablen angegeben als Anzahl (%) der Patienten pro Gruppe, kontinuierliche Variablen als Median [Spannweite] sofern nicht anders angegeben. *Dosing Adherence* (Anzahl der Tage mit korrekter Anzahl an OAT‐Einnahmen bezogen auf beobachtete Tage), *Taking Adherence (*Anzahl der Einnahmen im Verhältnis zu vorgeschriebenen Einnahmen), *Timing Adherence* (Anteil der Einnahmen innerhalb des vordefinierten Zeitintervalls von ± 3 Stunden), *Therapiebeginn* (I, Zeit zwischen erster geplanter und beobachteter Einnahme), *Drug Holidays* (Anzahl und Dauer vergessener Einnahmen für mindestens 48 Stunden bei OAT, die 1 x täglich eingenommen werden, oder 24 Stunden bei OAT, die 2 x täglich eingenommen werden), *Persistenz* (ungeplante Therapieabbrüche für ≥ 7 Tage).

^a^ Patienten, die mit OAT mit 1 x täglicher Einnahme behandelt wurden: n = 47.

^b^ Patienten, die mit OAT mit 2 x täglicher Einnahme behandelt wurden: n = 37.

^c^ Nur bei Patienten erfasst, die bei Therapiebeginn der OAT beraten wurden: n = 36.

**ABBILDUNG 4:**
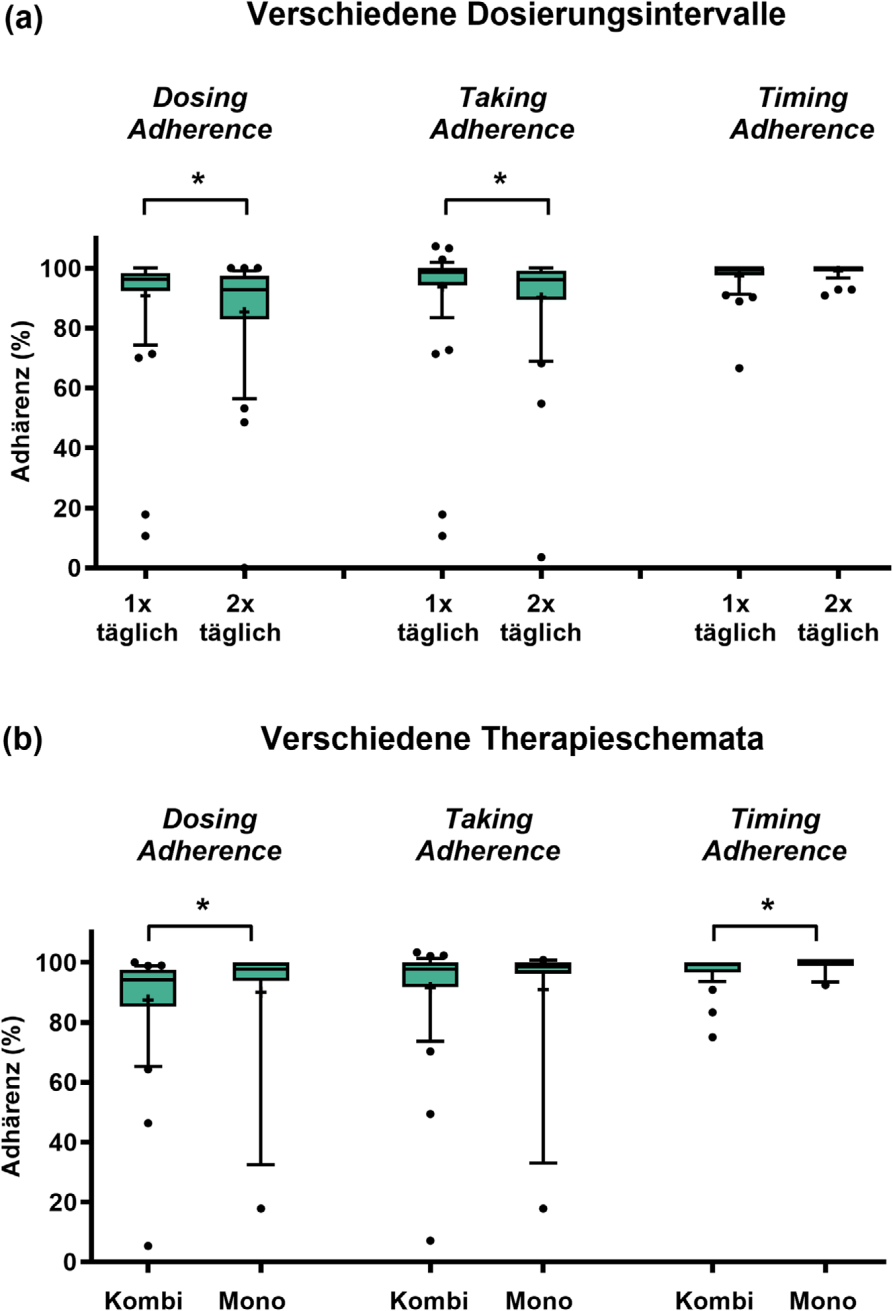
Einfluss verschiedener OAT‐Einnahmeschemata auf die Adhärenzparameter. *Dosing, Taking* und *Timing Adherence* gezeigt für (a) verschiedene Dosierungsintervalle und (b) verschiedene Therapieschemata. *Dosing Adherence (*Anzahl der Tage mit korrekter Anzahl von OAT‐Einnahmen bezogen auf beobachteten Tage), *Taking Adherence* (Anzahl der Einnahmen im Verhältnis zu vorgeschriebenen Einnahmen), *Timing Adherence* (Anteil der Einnahmen innerhalb des vordefinierten Zeitintervalls von ± 3 Stunden). Daten in Prozent, gemessen mit MEMS^®^ Buttons. Box‐Plots mit + als Mittelwert und Whiskers vom 10. bis 90. Perzentil, *p < 0,05 (Mann‐Whitney‐Test). Adhärenzdaten von zwei Patienten waren für Woche 0–12 nicht auswertbar (1 x Patient verstorben, 1 x Einwilligung widerrufen, zu kompliziert) und Adhärenzdaten von sechs Patienten waren für Woche 4–12 nicht auswertbar (3 x Einwilligung widerrufen, zu kompliziert, 1 x Patient verstorben, 1 x *lost to follow‐up* und 1 x Einwilligung widerrufen, kognitive Beeinträchtigung). *Abk*.: Kombi, OAT Kombinationstherapie; MEMS, Medication Event Monitoring System; Mono, OAT Monotherapie; OAT, orale Antitumortherapeutika

Insgesamt waren die mediane *DA* (96,4% vs. 92,8%; p = 0,0253) und die *Taking Adherence* (98,5% vs. 96,2%; p = 0,0127) bei OAT mit 1 x täglicher Einnahme höher als bei OAT mit 2 x täglicher Einnahme (Abbildung 4a). Die *Dosing*, *Taking*, und *Timing Adherence* änderte sich im Laufe der Zeit weder bei OAT mit 1 x noch mit 2 x täglicher Einnahme signifikant (Online‐Abbildung S2). Die *DA* war bei Patienten unter OAT‐Monotherapien signifikant höher als bei Kombinationsschemata (Abbildung 4b). Die Adhärenzparameter sind in der Online‐Abbildung S3a,b für verschiedene OAT stratifiziert dargestellt. Beim Vergleich von *Dosing*, *Taking* und *Timing Adherence* wurden keine Unterschiede zwischen Patienten, die die erste Beratung zu Therapiebeginn oder im Therapieverlauf erhielten, festgestellt (Online‐Abbildung S3c).

**ABBILDUNG 5:**
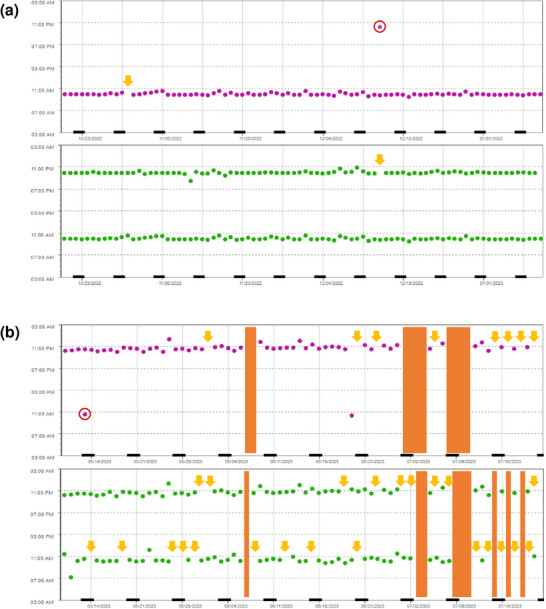
Ausgewählte Adhärenzprofile. Beispiele für: (a) einen adhärenten Patienten und (b) einen non‐adhärenten Patienten. Beide Patienten wurden mit einer Kombination aus OAT mit 1 x täglicher (lila Punkte) und 2 x täglicher Einnahme (grüne Punkte) behandelt. Rote Kreise: zusätzliche OAT‐Einnahmen durch den Patienten. Gelbe Pfeile: einzelne, ausgelassene OAT‐Einnahmen. Orangefarbene Balken: mehrfache, ausgelassene OAT‐Einnahmen (*D* *rug* *H* *olidays*). Daten gemessen mit MEMS^®^ Buttons, angegeben als Datum und Zeitpunkt der Medikamenteneinnahme (= MEMS^®^ Button gedrückt). *Abk*.: MEMS, Medication Event Monitoring System; OAT, orale Antitumortherapeutika

Insgesamt wurden sechs Patienten als non‐adhärent eingestuft (*DA* ≤ 80%). Abgesehen von einem jüngeren Durchschnittsalter (p = 0,0008) und einem höheren Anteil an berufstätigen Patienten (p = 0,0117) wurden keine Unterschiede zwischen non‐adhärenten und adhärenten Patienten festgestellt (Tabelle S4, Online Supplement). Beispiele für Adhärenzprofile sind in Abbildung 5a,b dargestellt.

### Empfehlungen für die Arzneimitteltherapiesicherheit

**ABBILDUNG 6:**
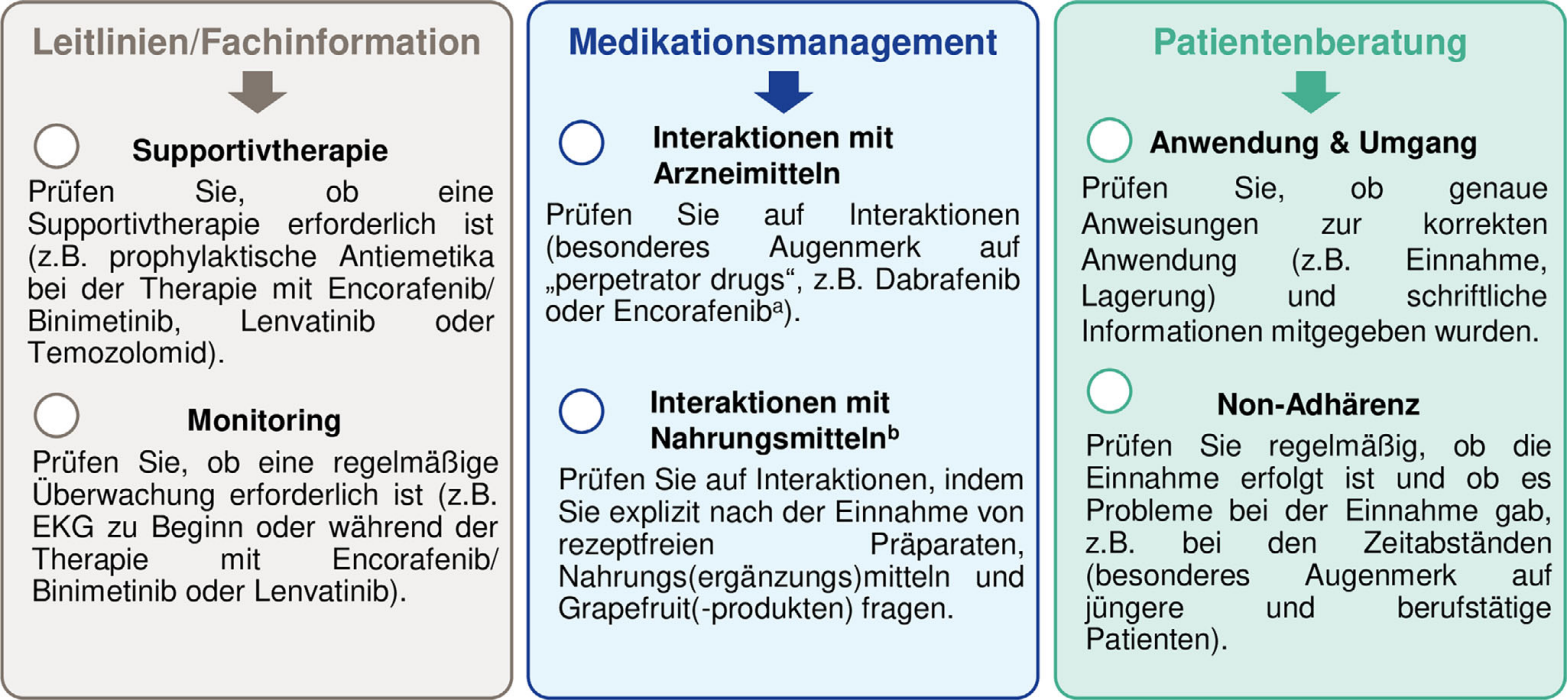
Top‐Empfehlungen zur Optimierung der Arzneimitteltherapiesicherheit bei OAT in der dermato‐onkologischen Patientenversorgung. *Abk*.: CYP, Cytochrom P450; EKG, Elektrokardiogramm; OAT, orale Antitumortherapeutika; OTC, *over‐the‐counter* (freiverkäuflich). ^a^Aufgrund neuer, nach dem Studienzeitraum veröffentlichter Daten, wird Encorafenib als starker CYP3A4‐Induktor eingestuft.^8^ ^b^Umfasst OTC‐Arzneimittel und Nahrungs(ergänzungs)mittel.

Auf Grundlage der *(1)* am häufigsten beobachteten Ursachen für OAT‐bezogene Medikationsfehler und *(2)* der Ergebnisse unseres Adhärenzmonitorings haben wir eine Checkliste zusammengestellt. Diese gezielten Empfehlungen können dazu beitragen, die Arzneimitteltherapiesicherheit bei OAT in der dermato‐onkologischen Patientenversorgung weiter zu optimieren (Abbildung 6).

## DISKUSSION

Wir führten umfassende Medikationsanalysen gemäß des evidenzbasierten AMBORA‐Therapiebegleitungskonzepts^9^ durch und erfassten sowohl die objektive als auch die subjektive Adhärenz gegenüber dermatologischer oraler Antitumortherapeutika (OAT) mit Hilfe von MEMS^®^ Buttons (Medication Event Monitoring System) und des MARS‐D‐Fragebogens. Zum einen stellten wir eine hohe Anzahl von 1,6 Medikationsfehlern pro Patient innerhalb der Gesamtmedikation fest, von denen fast zwei Drittel OAT‐bezogen waren (Abbildung 2) und zu 89% gelöst werden konnten. Zum anderen war die OAT‐Adhärenz (*Dosing Adherence*) über 12 Wochen hoch (95%).

Interaktionen zwischen Arznei‐ oder Nahrungs(ergänzungs)mitteln waren die häufigsten Ursachen für OAT‐bezogene Fehler (etwa 40%) (Abbildung 3). Daten von Patienten mit anderen Tumorentitäten, die in unserem AMBORA‐Zentrum beraten wurden, zeigten, dass Interaktionen zwischen Arznei‐ oder Nahrungs(ergänzungs)mitteln 24% aller Fehler ausmachten.^21^ Dies unterstreicht die Annahme, dass dermatologische OAT besonders interaktionsanfällig sind. Eine andere Studie bei Patienten mit verschiedenen Tumorentitäten zeigte, dass 14% der potenziellen Interaktionen auf OAT‐bezogene Interaktionen zurückzuführen sind.^22^ Diese Daten sind aufgrund heterogener Bewertungsmethoden nur bedingt vergleichbar. Sie weisen aber darauf hin, dass gezielte Strategien erforderlich sind, um diese Herausforderungen zu adressieren. So wird in der deutschen Fachinformation von Dabrafenib eine umfassende Medikationsanalyse vor Therapiebeginn empfohlen.^8^ Während die starke Cytochrom‐P450 (CYP)3A4‐Induktion durch Dabrafenib seit Jahren bekannt ist, wurde ein ähnliches oder sogar noch stärkeres Interaktionspotenzial für Encorafenib durch neue Daten belegt, die nach Abschluss unserer Untersuchung veröffentlicht wurden.^8^

Wir beobachteten einen hohen Anteil von Patienten, die rezeptfreie OTC‐Arzneimittel einschließlich Nahrungs(ergänzungs)mittel verwendeten (70%). Umfragen bei Melanompatienten ergaben, dass 27% von 100 Patienten angaben, komplementäre alternative Medizin (CAM) zu verwenden und nur 50% der Patienten ihre Ärzte darüber informierten.^23^ Daher sollte die Anwendung von CAM vor Beginn der Therapie ausdrücklich erfragt werden. Pharmazeuten mit onkologischer Expertise sind wichtige Akteure, um im Rahmen eines interprofessionellen Ansatzes Interaktionen zwischen Arznei‐ oder Nahrungs(ergänzungs)mitteln zu verhindern^24^ sowie Nebenwirkungen^25^ zu adressieren. Diese Daten unterstreichen die Notwendigkeit einer sorgfältigen Medikationsanalyse einschließlich rezeptfreier OTC‐Arzneimittel und Nahrungs[ergänzungs]mittel (zum Beispiel CAM) als eine unserer Schlüsselempfehlungen zur Optimierung der Arzneimitteltherapiesicherheit (Abbildung 6).

Insgesamt hatten 43% der OAT‐bezogenen Medikationsfehler patientenbezogene Ursachen. Neben anderen Interventionen gelten die Beratung durch klinische Pharmazeuten, die Patientenschulung und das Medikationsmanagement als erfolgreiche Maßnahmen zur Förderung der Adhärenz.^26^ Die Einbeziehung von Pharmazeuten in die Behandlungsteams wird in mehreren Untersuchungen empfohlen.^27^, ^28^

Insgesamt lagen die mediane objektive (95%, *DA*) und subjektive (100%) Adhärenz der im AMBORA‐Zentrum beratenen Patienten im oberen Bereich der in der Literatur berichteten Werte.^5^, ^6^ Daten zu anderen OAT sind jedoch nur eingeschränkt vergleichbar, da die Bewertungsmethoden variieren und nur wenige Studien sowohl objektive als auch subjektive Verfahren einsetzen. Bei einer Untersuchung von Patienten mit soliden Tumoren (gastrointestinale Tumorerkrankungen und Brustkrebs) und hämatologischen Erkrankungen wurden für die jeweiligen Populationen Adhärenzraten von 85% und 94% sowie eine subjektive Adhärenz von 86% und 95% detektiert.^29^ Die Untersuchung wurde im Rahmen eines von Pharmazeuten geleiteten OAT‐Managementprogramms über 12 Wochen durchgeführt.^29^

Marin et al. verwendeten MEMS^®^ Caps, Selbstberichte und die Zählung von Tabletten, um die Adhärenz bei Patienten zu messen, die 1 x täglich mit Imatinib behandelt wurden: Nach circa 3 Monaten lag die mit den MEMS^®^ Caps gemessene Adhärenz im Median bei 98% und 14% der Patienten wiesen Adhärenzraten ≤ 80% auf.^7^ Selbstberichte und Tablettenzählungen überschätzen die Adhärenz eher.^7^ In einer anderen Studie wurde die Adhärenz von Capecitabin bei Patienten mit Brustkrebs und Kolorektalkarzinom im Rahmen eines pharmazeutischen Therapiebegleitungskonzepts über etwa vier Monate mit den MEMS^®^ Caps gemessen und eine vergleichbare mittlere *DA* von 97% ermittelt.^30^

In Übereinstimmung mit anderen Veröffentlichungen^31^, ^32^ beobachteten wir eine geringere Adhärenz bei OAT mit 2 x täglicher Einnahme im Vergleich zu 1 x täglicher Einnahme (Abbildung 4). Angesichts des vorherrschenden Einsatzes von Kombinationstherapien mit BRAF/MEK‐Inhibitoren könnten Melanompatienten als besonders anfällig für Non‐Adhärenz gelten. Trotz des intensivierten AMBORA‐Therapiebegleitungskonzepts wurden sechs Patienten als non‐adhärent identifiziert (Abbildung 5b). Wir konnten feststellen, dass die Adhärenz insbesondere bei jüngeren Patienten ein Problem darstellen kann, was zum Beispiel bereits bei Brustkrebs‐Patientinnen^33^ oder bei Psoriasis‐Patienten, die eine systemische Behandlung erhielten^34^, beschrieben wurde. Während die Diagnose und anschließende Behandlung mit OAT beim Basalzellkarzinom oder kutanem T‐Zell‐Lymphom eher auf ein höheres Alter beschränkt ist, werden OAT beim Melanom zunehmend in früheren Therapielinien eingesetzt, die auch jüngere Patienten einschließen (zum Beispiel adjuvante Therapien).^35^ In einer systematischen Übersichtsarbeit lag die Adhärenz bei der adjuvanten hormonellen Brustkrebstherapie nach 5 Jahren zwischen 41% und 72%, wobei 31% bis 73% die Behandlung abbrachen.^36^ Non‐Adhärenz und Therapieabbrüche wurden in diesem Zusammenhang mit einer erhöhten Sterblichkeit in Verbindung gebracht.^37^ Daher sollten individuelle Umstände (zum Beispiel Alter, Berufsleben) in interprofessionellen Therapiebegleitungskonzepten gezielt adressiert werden (Abbildung 6).

Wir betrachten die kombinierte Erfassung der objektiven und subjektiven Adhärenz als eine der größten Stärken unserer Untersuchung. Validierte, subjektive Fragebögen sind zwar eine verbreitete und praktikable Methode zur Identifizierung von Non‐Adhärenz, ihre Zuverlässigkeit ist jedoch oft nicht überzeugend und sie könnten die Adhärenz im Vergleich zu objektiven Methoden überschätzen.^7^, ^26^ Darüber hinaus basierte unser Therapiebegleitungskonzept auf SOP, die in der AMBORA‐Studie^9^ etabliert wurden und die Validität sicherstellen. Durch die Beratung der Patienten und die Durchführung des Adhärenzmonitorings in der dermato‐onkologischen Routineversorgung konnten wir Patienten einer repräsentativen Untergruppe unabhängig von der Behandlungsdauer einbeziehen (Tabelle S3, Online‐Supplement).

Dermato‐onkologische Patienten werden häufig simultan mit einer Kombination aus OAT mit 1 x und 2 x täglicher Einnahme behandelt. Die Untersuchung der Adhärenz in dieser Kohorte ermöglichte es uns, die Adhärenz beider Schemata bei ein und demselben Patienten zu vergleichen und so alle patientenspezifischen Faktoren auszuschließen, welche die Adhärenz beeinflussen könnten.

Folgende Limitationen unserer Arbeit sind zu berücksichtigen: Aus ethischen Gründen haben wir eine nicht‐randomisierte Untersuchung durchgeführt. Wir würden erwarten, dass die Arzneimitteltherapiesicherheit und Adhärenz in der klinischen Routine ohne das AMBORA‐Therapiebegleitungskonzept niedriger ist. Die Verwendung der MEMS^®^ Buttons stellt lediglich ein Surrogat für die Medikamenteneinnahme dar und weder die tatsächliche Anzahl der eingenommenen Tabletten/Kapseln noch das Zeitintervall zwischen Medikamenten‐ und Nahrungseinnahme konnte erfasst werden. Dadurch könnte die Adhärenz unterschätzt worden sein. Ein *T* *herape* *u* *ti* *sches* *Drug Monitoring* ^38^, ^39^ wäre eine Möglichkeit, diese Verzerrung auszugleichen. Die Aussagekraft wäre jedoch begrenzt, da Patienten unter Umständen nur an den Tagen vor der Blutentnahme adhärent gewesen sein könnten.

Die Intervention selbst könnte darüber hinaus die Adhärenz gefördert haben, insbesondere in den ersten Wochen der Therapie. Interessanterweise wurden beim Vergleich der Adhärenzraten über die Zeit jedoch keine Unterschiede festgestellt (Abbildungen S1, S2; Online‐Supplement). Darüber hinaus konnte Non‐Adhärenz während des Studienzeitraums nicht direkt adressiert werden, da die Adhärenzprofile erst retrospektiv ausgewertet wurden. Zukünftige Arbeiten sollten eine proaktive Beratung auf Grundlage von Adhärenzprofilen umfassen, ähnlich einer kürzlich durchgeführten Studie bei Patienten, die mit verschiedenen OAT behandelt wurden.^40^

Zusammenfassend traten Medikationsfehler bei der Behandlung mit dermatologischen OAT häufig auf und der Großteil konnte im AMBORA‐Zentrum behoben werden. Sowohl die objektive als auch die subjektive OAT‐Adhärenz war hoch. Besonderes Augenmerk sollte auf die Adhärenz bei OAT mit 2 x täglicher Einnahme sowie bei jüngeren, noch berufstätigen Patienten gelegt werden. Unsere Daten unterstreichen den Nutzen einer interprofessionellen Zusammenarbeit unter Einbeziehung von klinischen Pharmakologen/Pharmazeuten. Die abgeleiteten, gezielten Empfehlungen für die dermato‐onkologische Routineversorgung können zur Optimierung der Arzneimitteltherapiesicherheit beitragen.

## FÖRDERUNG

Diese Arbeit wurde von der Deutschen Krebshilfe (DKH; Projektnummer 70114066/70114067) unterstützt. Die Drittmittelgeber hatten keinen Einfluss auf das Design, die Datenerhebung, die Analyse oder die Interpretation.

## DANKSAGUNG

Open access Veröffentlichung ermöglicht und organisiert durch Projekt DEAL.

## INTERESSENKONFLIKT

L.C. erklärt, dass kein Interessenkonflikt besteht. F.D. erhielt Beratungshonorare von Lilly und Sandoz‐Hexal, Vortragshonorare von Johnson & Johnson und Gilead sowie eine zweckgebundene finanzielle Zuwendung (erster Preis des MSD Gesundheitspreises Deutschland 2021). R.K. erhielt Vortragshonorare von Pierre Fabre sowie Unterstützung für Reisen/Teilnahmen an wissenschaftlichen Veranstaltungen von Pierre Fabre, Novartis und SUN Pharmaceuticals. P.D. erhielt Vortragshonorare von AstraZeneca GmbH sowie eine zweckgebundene finanzielle Zuwendung (erster Preis des MSD Gesundheitspreises Deutschland 2021). M.E. erhielt Vortragshonorare von Immunocore, Novartis, Pierre Fabre und Sanofi, Unterstützung für Reisen/Teilnahmen an wissenschaftlichen Veranstaltungen von Novartis und Pierre Fabre sowie eine Tätigkeit im Data Safety Monitoring Board/Advisory Board von Sanofi. M.F.F. erhielt Fördermittel oder Forschungsaufträge von Boehringer Ingelheim und Heidelberg Pharma Research GmbH, Beratungshonorare und Vortragshonorare von Boehringer Ingelheim sowie eine zweckgebundene finanzielle Zuwendung (erster Preis des MSD Gesundheitspreises Deutschland 2021). C.B. erhielt Beratungshonorare von Almirall Hermal, BMS, Delcath, Immunocore, MSD, Novartis, Pierre Fabre, Regeneron und Sanofi, Vortragshonorare von Almirall Hermal, BMS, Leo Pharma, MSD, Novartis und Pierre Fabre, Unterstützung für Reisen/Teilnahmen an wissenschaftlichen Veranstaltungen von Pierre Fabre sowie eine Tätigkeit im Data Safety Monitoring Board/Advisory Board von Miltenyi und InflaRx. K.G. erhielt Vortragshonorare von AstraZeneca GmbH und Roche Pharma sowie eine zweckgebundene finanzielle Zuwendung (erster Preis des MSD Gesundheitspreises Deutschland 2021).

## Supporting information



Supplementary information
