# Supplementary material for: Auswirkungen auf die Arzneimitteltherapiesicherheit und Adhärenz in der Dermato‐Onkologie: Das AMBORA‐Therapiebegleitungskonzept für orale Antitumortherapeutika
Source: J Dtsch Dermatol Ges. 2025 Oct 23;23(10):1245–58. [Article in German] doi: 10.1111/ddg.15809_g (PMC12548321; doi:10.1111/ddg.15809_g)
Supplement: Supplementary file 1 — Supplementary information [file DDG-23-1245-s001.pdf]

# ONLINE SUPPLEMENT

*Cuba L et al. Auswirkungen auf die Arzneimitteltherapiesicherheit und Adhärenz in der Dermato-Onkologie: Das AMBORA-Therapiebegleitungskonzept für orale Antitumortherapeutika*

**ONLINE SUPPLEMENT: METHODIK** .................................................................. **2**

**ONLINE SUPPLEMENT: ABBILDUNGEN** .............................................................. **4**

ABBILDUNG S1 „*Dosing Adherence*“ und subjektive Adhärenz über die Zeit. ....... 4

ABBILDUNG S2 Einfluss verschiedener Dosierungsintervalle auf die Adhärenzparameter über die Zeit. ........................................................................ 5

ABBILDUNG S3 Einfluss verschiedener OAT-Therapieschemata oder Zeitpunkte der OAT-Behandlung auf die Adhärenzparameter. ................................................ 6

**ONLINE SUPPLEMENT: TABELLEN** .................................................................. **8**

TABELLE S1 Charakteristika der Patienten, die im AMBORA-Zentrum beraten und mit dermatologischen OAT behandelt wurden - stratifiziert nach Erstberatung bei Therapiebeginn oder im Therapieverlauf. ............................................................... 8

TABELLE S2 Charakteristika der Patienten, die im AMBORA-Zentrum beraten und mit dermatologischen OAT behandelt wurden - stratifiziert nach Teilnahme am Adhärenzmonitoring. ......................................................................................... 10

TABELLE S3 Ausgewählte Beispiele für OAT-bezogene Medikationsfehler und deren Ursachen. ........................................................................................ 12

TABELLE S4 Charakteristika der Patienten, die im AMBORA-Zentrum beraten und mit dermatologischen OAT behandelt wurden - stratifiziert nach Adhärenz. ......... 14

# ONLINE SUPPLEMENT: METHODIK

## (1) Bewertung von Medikationsfehlern

Umfassende Medikationsanalyse wurden gemäß der Definition des „Pharmaceutical Care Network Europe“ (PCNE) durchgeführt[1]. Zur Bewertung wurden die Fachinformationen der Arzneimittel und andere evidenzbasierte Quellen herangezogen: z.B. Datenbanken für Interaktionen zwischen Arznei- und Nahrungs(ergänzungs)mitteln[2-4] oder Leitlinien zur Supportivtherapie (z.B. Antiemese[5]).

## (2) Adhärenzmonitoring

Die MEMS® Buttons,[6] erinnerten die Patienten nicht aktiv an die Einnahme der OAT. Für jedes OAT wurde nach erfolgter Einweisung ein separater Button ausgehändigt (d.h. zwei Buttons für Kombinationstherapien), der an der Arzneimittelverpackung befestigt werden konnte. Die Zeitpunkte der Einnahme wurden erfasst und nach Rückgabe der Buttons retrospektiv ausgewertet. Zur Dokumentation von technischen Problemen oder Einnahmefehlern (z.B. korrekte OAT-Einnahme, aber vergessenes Drücken der Buttons) wurde ein Formular zur Verfügung gestellt. Diese Ereignisse wurden nicht als vergessene Einnahmen gezählt.

### Adhärenzparamenter

- *„Dosing Adherence“ = Anzahl der Tage mit korrekter Anzahl von OAT-Einnahmen bezogen auf die beobachteten Tage*
- *„Taking Adherence“ = Anzahl der Einnahmen im Verhältnis zu den vorgeschriebenen Einnahmen*
- *„Timing Adherence“ = Anteil der Einnahmen innerhalb des vordefinierten Zeitintervalls von ±3 Stunden*
- *„Therapiebeginn“ (Zeit zwischen der ersten geplanten und der beobachteten Einnahme*
- *„Drug Holidays“ (Anzahl und Dauer der vergessenen Einnahmen für mindestens 48 Stunden bei OAT mit 1x täglicher Einnahme werden bzw.24 Stunden bei OAT mit 2x täglicher Einnahme)*
- *„Persistenz“ (ungeplante Therapieabbrüche für ≥7 Tage)*

**Referenzen**


1. Pharmaceutical Care Network Europe (PCNE) position paper on medication review (2016). Zugriff über: https://www.pcne.org/upload/files/149_Position_Paper_on_PCNE_Medication_Review_final.pdf (letzter Zugriff am 30.01.2024).
2. UpToDate®. Drug interactions. Zugriff über: https://www.uptodate.com/contents/search (letzter Zugriff am 30.01.2024).
3. Pharmaceutical press: Stockley's interactions checker. Zugriff über: https://www.medicinescomplete.com/log-in/ (letzter Zugriff am 30.01.2024).
4. Memorial Sloan Kettering Cancer Center (MSKCC): Search about herbs. Zugriff über: https://www.mskcc.org/cancer-care/diagnosis-treatment/symptom-management/integrative-medicine/herbs/search (letzter Zugriff am 30.01.2024).
5. National Comprehensive Cancer Network (NCCN) clinical practice guidelines in oncology: Antiemesis. Version 1.2024, 13. Dezember 2023. Zugriff über: https://www.nccn.org/professionals/physician_gls/pdf/antiemesis.pdf (letzter Zugriff am 04.06.2024).
6. AARDEX® Group. MEMS® Button ('Medication Event Monitoring System'). Zugriff über: https://aardexgroup.com/medication-event-monitoring-system/ (letzter Zugriff am 04.06.2024)

# ONLINE SUPPLEMENT: ABBILDUNGEN

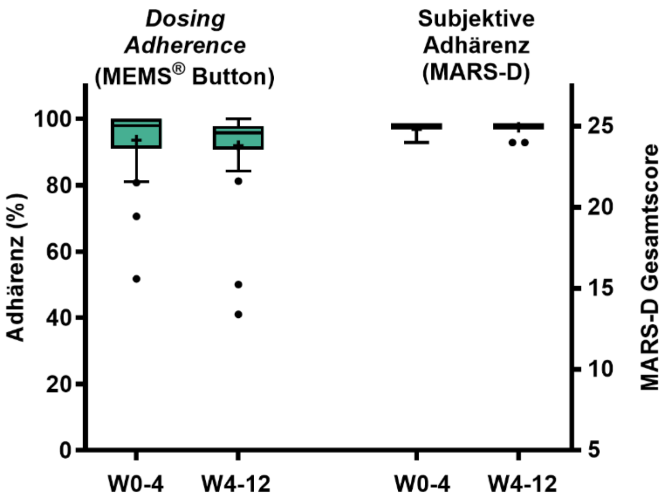


**ABBILDUNG S1 „*Dosing Adherence*“** und subjektive Adhärenz über die Zeit.

Adhärenzparameter stratifiziert nach Woche 0 bis 4 und Woche 4 bis 12 der Teilnahme am Adhärenzmonitoring. „Dosing Adherence“ in %, gemessen mit MEMS® Buttons. Subjektive Adhärenz in % des Gesamtscores (5-25), gemessen mit dem MARS-D Fragebogen. Box-Plots mit + als Mittelwert und Whiskers vom 10. bis 90. Perzentil (Wilcoxon-Vorzeichen-Rang-Test).

MARS-D, Medication Adherence Reporting Scale, validierte deutsche Übersetzung; MEMS, Medication Event Monitoring System; W, Woche.

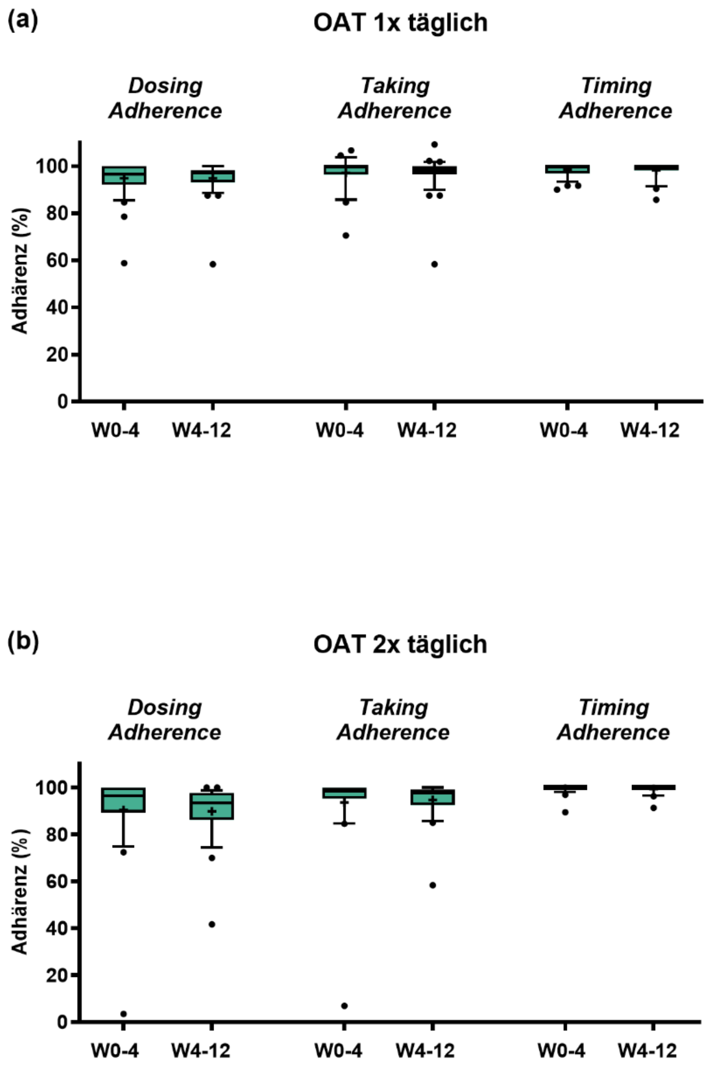


**ABBILDUNG S2** Einfluss verschiedener Dosierungsintervalle auf die Adhärenzparameter über die Zeit.

„*Dosing*", „*Taking*", und „*Timing Adherence*" gezeigt für OAT mit **(a)** 1x täglicher und **(b)** 2x täglicher Einnahme. Adhärenzparameter stratifiziert nach Woche 0 bis 4 und Woche 4 bis 12 der Teilnahme am Adhärenzmonitoring. Daten in %, gemessen mit den MEMS® Buttons. Box-Plots mit + als Mittelwert und Whiskers von der 10. bis 90. Perzentile (Wilcoxon-Vorzeichen-Rang-Test).

MEMS, Medication Event Monitoring System; OAT, orale Antitumortherapeutika; W, Woche.

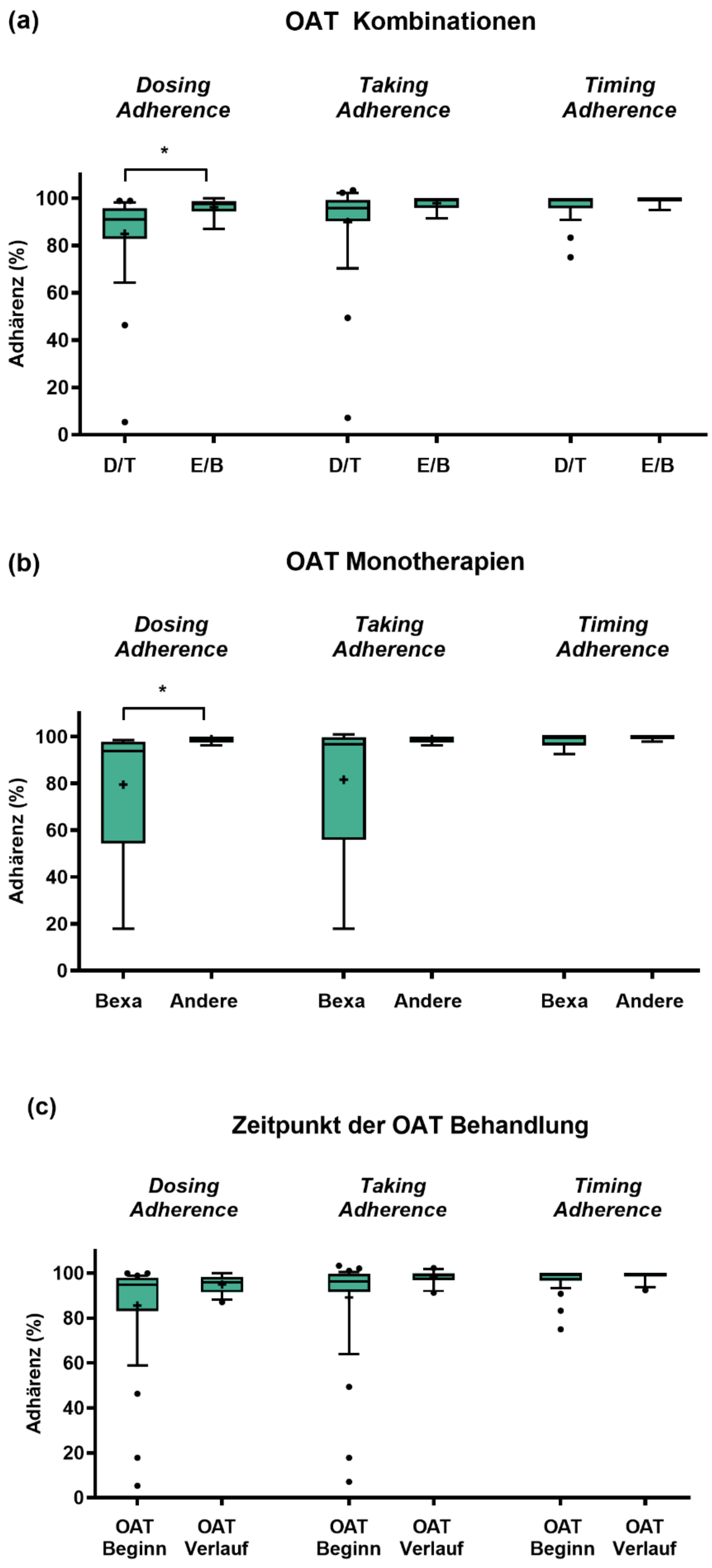


**ABBILDUNG S3** Einfluss verschiedener OAT-Therapieschemata oder Zeitpunkte der OAT-Behandlung auf die Adhärenzparameter.

„*Dosing*”, „*Taking*” und „*Timing Adherence*” gezeigt für **(a)** verschiedene OAT-Kombinationen, **(b)** verschiedenen OAT-Monotherapien und **(c)** Erstberatung zu

Therapiebeginn oder im Therapieverlauf. Daten in %, gemessenmit den MEMS® Buttons. Box-Plots mit + als Mittelwert und Whiskern von der 10. bis 90. Perzentile, *p < 0,05 (Mann-Whitney-Test).

Bexa, Bexaroten; D/T, Dabrafenib/Trametinib; E/B, Encorafenib/Binimetinib; MEMS, Medication Event Monitoring System; OAT, orale Antitumortherapeutika.

# ONLINE SUPPLEMENT: TABELLEN

**TABELLE S1** Charakteristika der Patienten, die im AMBORA-Zentrum beraten und mit dermatologischen OAT behandelt wurden - stratifiziert nach Erstberatung bei Therapiebeginn oder im Therapieverlauf.

| | **Anzahl an Patienten (%)** | | |
|---|---|---|---|
| | **OAT Therapiebeginn *n*=57** | **OAT Therapieverlauf *n*=35** | ***p*** |
| **Patientencharakteristika** | | | |
| Alter, Jahre (Mittelwert) | 62,4 [29-90] | 59,0 [31– 88] | *ns* |
| Weibliches Geschlecht | 34 (59,6) | 18 (51,4) | *ns* |
| ECOG 0-1 | 39 (68,4) | 27 (77,1) | *ns* |
| ECOG >1 | 18 (31,6) | 8 (22,9) | |
| Berufstätig | 12 (21,1) | 10 (28,6) | *ns* |
| Unterstützung im Alltag benötigt | 11 (19,3) | 6 (17,1) | *ns* |
| Grapefruitkonsum | 8 (14,0) | 4 (11,4) | *ns* |
| Einnahme von ≥1 OTC-Arzneimitteln[a] | 39 (68,4) | 25 (71,4) | *ns* |
| **Medikation pro Patient** (Median) | | | |
| Gesamtmedikation[b] | 9 [1-21] | 7 [3-22] | *ns* |
| Orale Antitumortherapeutika[c] | 2 [1-3] | 2 [1-3] | *ns* |
| Begleitmedikation | 7 [0-20] | 6 [1-20] | *ns* |
| OTC-Arzneimittel[a] | 1 [0-10] | 1 [0-5] | *ns* |
| **Lebenssituation** | | | |
| Mit Partner/Familie | 43 (75,4) | 28 (80,0) | *ns* |
| Alleinstehend | 11 (19,3) | 5 (14,3) | |
| In Pflegeeinrichtung | 3 (5,3) | 1 (2,9) | |
| NA | – | 1 (2,9) | |
| **Tumorart** | | | |
| Melanom | 48 (84,2) | 27 (77,1) | *ns* |
| Kutanes T-Zell Lymphom | 6 (10,5) | 5 (14,3) | |
| Basalzellkarzinom | 3 (5,3) | 3 (8,6) | |
| **Orale Antitumortherapeutika[d]** | | | |
| Dabrafenib/Trametinib | 35 (61,4) | 14 (40,0) | *ns* |
| Encorafenib/Binimetinib | 7 (12,3) | 11 (31,4) | |
| Bexaroten | 5 (8,8) | 5 (14,3) | |
| Temozolomid | 3 (5,3) | 1 (2,9) | |
| Vemurafenib/Cobimetinib | 2 (3,5) | 1 (2,9) | |
| Sonidegib | 1 (1,8) | 2 (5,7) | |
| Vismodegib | 2 (3,5) | 1 (2,9) | |
| Lenvatinib | 1 (1,8) | – | |
| Acitretin | 1 (1,8) | – | |
| **Behandlungscharakteristika** | | | |
| Zyklische Einnahme | 5 (8,8) | 3 (8,6) | *ns* |
| Kurativ/adjuvant | 16 (28,1) | 7 (20,0) | *ns* |
| Off-label-Einsatz[e] | 5 (8,8) | 3 (8,6) | *ns* |

Abkürzungen: ECOG, Eastern Cooperative Oncology Group; NA, nicht zutreffend; OAT, orale Antitumortherapeutika; OTC, “over-the-counter” (freiverkäuflich). *Anmerkung:* Die Charakteristika gelten für den Beginn der Untersuchung (Zeitpunkt der ersten Beratung). Kategorische Variablen angegeben als Anzahl (%) der Patienten pro Gruppe, kontinuierliche Variablen als Mittelwert oder Median [Spannweite].

ns, nicht signifikant (t-Test, Mann-Whitney-Test, Chi$^2$-Test oder Fishers’ Exact Test).

[a]Umfasst OTC-Arzneimittel und Nahrungs(ergänzungs)mittel.

[b]Umfasst Arzneimittel aller Applikationsarten (z.B. oral, parenteral oder topisch) und OTC-Arzneimittel, sowie Nahrungs(ergänzungs)mittel.

[c]Zwei Patientinnen mit Melanom, die mit Dabrafenib/Trametinib behandelt wurde, wurde außerdem Exemestan bzw. Talazoparib bei Brustkrebs verordnet. Ein Patient wurde mit Bexaroten und Methotrexat behandelt.

[d]Umfasst nur OAT, die für dermato-onkologische Indikationen verschrieben wurden.

[e]Umfasst die palliative Therapie mit Temozolomid, Lenvatinib, Acitretin und die adjuvante Therapie mit Encorafenib/Binimetinib.

**TABELLE S2** Charakteristika der Patienten, die im AMBORA-Zentrum beraten und mit dermatologischen OAT behandelt wurden - stratifiziert nach Teilnahme am Adhärenzmonitoring.

| | **Anzahl an Patienten (%)** | | |
|---|---|---|---|
| | **Teilnahme am Adhärenzmonitoring *n*=52** | **Andere *n*=40** | ***p*** |
| **Patientencharakteristika** | | | |
| Alter, Jahre (Mittelwert) | 61,6 [29-90] | 60,4 [31-88] | *ns* |
| Weibliches Geschlecht | 29 (55,8) | 23 (57,5) | *ns* |
| ECOG 0-1 | 37 (71,2) | 29 (72,5) | *ns* |
| ECOG >1 | 15 (28,8) | 11 (27,5) | |
| Berufstätig | 13 (25,0) | 9 (22,5) | *ns* |
| Unterstützung im Alltag benötigt | 10 (19,2) | 7 (17,5) | *ns* |
| Grapefruitkonsum | 9 (17,3) | 3 (7,5) | *ns* |
| Einnahme von ≥1 OTC-Arzneimitteln | 38 (73,1) | 26 (65,0) | *ns* |
| **Medikation pro Patient** (Median) | | | |
| Gesamtmedikation[b] | 9 [1-16] | 7 [2-22] | *ns* |
| Orale Antitumortherapeutika[c] | 2 [1-2] | 2 [1-3] | *ns* |
| Begleitmedikation | 7 [0-15] | 6 [0-20] | *ns* |
| OTC-Arzneimittel[a] | 1 [0-9] | 1 [0-10] | *ns* |
| **Lebenssituation** | | | |
| Mit Partner/Familie | 43 (82,7) | 28 (70,0) | *ns* |
| Alleinstehend | 8 (15,4) | 8 (20,0) | |
| In Pflegeeinrichtung | 1 (1,9) | 3 (7,5) | |
| NA | – | 1 (2,5) | |
| **Tumorart** | | | |
| Melanom | 44 (84,6) | 31 (77,5) | *ns* |
| Kutanes T-Zell Lymphom | 6 (11,5) | 5 (12,5) | |
| Basalzellkarzinom | 2 (3,8) | 4 (10,0) | |
| **Orale Antitumortherapeutika[d]** | | | |
| Dabrafenib/Trametinib | 32 (61,5) | 17 (42,5) | *ns* |
| Encorafenib/Binimetinib | 9 (17,3) | 9 (22,5) | |
| Bexaroten | 5 (9,6) | 5 (12,5) | |
| Temozolomid | 2 (3,8) | 2 (5,0) | |
| Vemurafenib/Cobimetinib | – | 3 (7,5) | |
| Sonidegib | 1 (1,9) | 2 (5,0) | |
| Vismodegib | 1 (1,9) | 2 (5,0) | |
| Lenvatinib | 1 (1,9) | – | |
| Acitretin | 1 (1,9) | – | |
| **Behandlungscharakteristika** | | | |
| Zyklische Einnahme | 2 (3,8) | 6 (15,0) | *ns* |
| Kurativ/adjuvant | 13 (25,0) | 13 (32,5) | *ns* |
| Off-label-Einsatz[e] | 5 (9,6) | 3 (7,5) | *ns* |

Abkürzungen: ECOG, Eastern Cooperative Oncology Group; NA, nicht zutreffend; OAT, orale Antitumortherapeutika; OTC, “over-the-counter” (freiverkäuflich). *Anmerkung:* Die Charakteristika gelten für den Beginn der Untersuchung (Zeitpunkt der ersten Beratung). Kategorische Variablen angegeben als Anzahl (%) der Patienten pro Gruppe, kontinuierliche Variablen als Mittelwert oder Median [Spannweite].

ns, nicht signifikant (t-Test, Mann-Whitney-Test, $Chi^2$-Test oder Fishers’ Exact Test).

[a]Umfasst OTC-Arzneimittel und Nahrungs(ergänzungs)mittel.

[b]Umfasst Arzneimittel aller Applikationsarten (z.B. oral, parenteral oder topisch) und OTC-Arzneimittel, sowie Nahrungs(ergänzungs)mittel.

[c]Zwei Patientinnen mit Melanom, die mit Dabrafenib/Trametinib behandelt wurde, wurde außerdem Exemestan bzw. Talazoparib bei Brustkrebs verordnet. Ein Patient wurde mit Bexaroten und Methotrexat behandelt.

[d]Nur OAT, die für dermato-onkologische Indikationen verschrieben wurden, werden angeführt.

[e]Umfasst die palliative Therapie mit Temozolomid, Lenvatinib, Acitretin und die adjuvante Therapie mit Encorafenib/Binimetinib.

**TABELLE S3** Ausgewählte Beispiele für OAT-bezogene Medikationsfehler und deren Ursachen.

| PCNE V9.1 | | Ursachen der OAT- bezogenen Medikationsfehler | Ausgewählte Beispiele |
|---|---|---|---|
| Verordnung & Arzneimittelauswahl | 1.3 | Interaktion (*Arzneimittel*) | **Dabrafenib** (moderater CYP3A4 Induktor) → Exemestan↓, Simvastatin↓ oder Sirolimus↓; **Bexaroten** (moderater CYP3A4 Induktor) → ↓ Atorvastatin |
| | 1.5 | Indikation ohne Arzneimittel | Keine prophylaktische Antiemese bei OAT mit moderat/hoch emetogenem Potential (z.B:.**Encorafenib/Binimetinib** oder **Temozolomid**) |
| | 3.1 | Dosierung zu niedrig | Dosierung von **Dabrafenib/Trametinib** um 50% zu niedrig bei Patienten mit Melanom mit Hirnmetastasen und hoher Symptomlast |
| | 3.4 | Dosierungsintervall zu häufig | **Bexaroten** 2x täglich oder 4x täglich verschrieben statt 1x täglich (Risiko für Non-Adhärenz ↑) |
| | 4.1 | Therapiedauer zu kurz | Folgeverordnungen für **Dabrafenib/Trametinib**, **Bexaroten** oder **Vismodegib** nicht zeitgerecht erfolgt |
| | 4.2 | Therapiedauer zu lang | **Lenvatinib** bei elektiver Operation nicht vorübergehend pausiert; Supportivmedikation (z.B. Fenofibrat) bei permanentem Therapieabbruch von **Bexaroten** nicht beendet |
| Abgabe | 5.1 | Verordnetes Arzneimittel nicht verfügbar | Bestellprobleme in öffentlichen Apotheken bei **Dabrafenib/Trametinib** oder **Sonidegib** |
| | 5.2 | Notwendige Information nicht oder nicht korrekt zur Verfügung gestellt | Einnahmehinweise für **Encorafenib/Binimetinib** durch Heilberufler oder Pflegepersonal nicht zur Verfügung gestellt |
| | 5.4 | Falsches Arzneimittel abgegeben | **Dabrafenib** und **Trametinib** bei Vorbereitung durch das Pflegepersonal verwechselt |
| Anwendung | 7.1 | Patient wendet Arzneimittel gar nicht oder weniger als verordnet an | Non-Adhärenz ohne ärztliche Rücksprache (z.B. Therapieabbruch von **Bexaroten** oder Dosisreduktionen von **Dabrafenib/Trametinib**) |
| | 7.2 | Patient wendet Arzneimittel mehr als verordnet an | **Trametinib** Einnahme 2x täglich statt 1x täglich |
| | 7.5 | Interaktion [*Nahrungsmittel*][a] | Grapefruit (moderater CYP3A4 Inhibitor) → ↑ **Dabrafenib,** ↑ **Encorafenib**; **Bexaroten** (Retinoid) ↔ Vitamin A; **Vemurafenib** (moderater CYP1A2 Inhibitor) → ↑ Koffein |
| | 7.6 | Patient bewahrt Arzneimittel falsch auf | **Dabrafenib** nicht bei Raumtemperatur sondern im Kühlschrank gelagert; Hände nach Umgang mit **Encorafenib/Binimetinib** nicht gewaschen trotz Enkel im Haushalt |
| | 7.7 | Ungeeigneter Einnahmezeitpunkt oder ungeeignetes Dosierungsintervall | Falscher Einnahmeabstand zur Nahrung bei **Sonidegib** (1 Stunde nach/2 Stunden vor dem Essen statt1 Stunde vor/2 Stunden nach dem Essen; Dosierungsintervall von **Binimetinib** 8 bzw. 16 Stunden statt alle 12 Stunden |
| | 7.8 | Patient wendet Arzneimittel unbeabsichtigt falsch an | Einnahme von **Dabrafenib/Trametinib** mit Nahrung statt nüchtern; Trinken von 1 Glas Wasser pro Tablette/Kapsel von **Encorafenib/Binimetinib** |
| | 7.10 | Patient ist nicht in der Lage, Anweisungen richtig zu verstehen | Patient mit **Dabrafenib/Trametinib** ist sich trotz Schulungsmaßnahmen nicht der Bedeutung von Sonnenschutz für die Haut bewusst |

| | | | |
|---|---|---|---|
| Sonstiges | 8.1 | Problem beim Medikationsabgleich | Dokumentierte Dosis von **Dabrafenib** 75mg 2-0-2, tatsächliche Dosis 1-0-1 wegen Niereninsuffizienz ; Einnahmeschema von **Temozolomid** an Tag 1-5 gefolgt von 21 Tagen statt 23 Tagen Pause |
| | 9.1 | Kein oder unzureichendes Monitoring (inkl. TDM) | Kein EKG vor Therapiebeginn oder im Therapieverlauf von **Encorafenib/Binimetinib** oder **Lenvatinib** |

Abkürzungen: ↑, erhöhte Toxizität; ↓, verringerte Effektivität; →, „perpetrator drug" (verursachender Arzneistoff) → „victim drug" (betroffener Arzneistoff); ↔, additiver Effekt; CYP, Cytochrom P450 Enzym; EKG, Elektrokardiogramm; OAT, orale Antitumortherapeutika; PCNE, Pharmaceutical Care Network Europe; TDM, Therapeutisches Drug Monitoring.

*Anmerkung:* OAT sind fett dargestellt.

[a]Umfasst OTC-Arzneimittel und Nahrungs(ergänzungs)mittel.

**TABELLE S4** Charakteristika der Patienten, die im AMBORA-Zentrum beraten und mit dermatologischen OAT behandelt wurden - stratifiziert nach Adhärenz.

| | Anzahl an Patienten (%) | | |
|---|---|---|---|
| | **Adhärent** ***n*=42** | **Nonadhärent** ***n*=6** | ***p*** |
| **Patientencharacteristika** | | | |
| Alter, Jahre (Mittelwert) | 64,2 [31-90] | 44,5 [29-59] | *** |
| Weibliches Geschlecht | 24 (57,1) | 4 (66,7) | *ns* |
| ECOG 0-1 | 29 (69,0) | 6 (100,0) | *ns* |
| ECOG >1 | 13 (31,0) | – | |
| Grapefruitkonsum | 7 (16,7) | 1 (16,7) | *ns* |
| Einnahme von ≥1 OTC-Arzneimitteln[a] | 31 (73,8) | 5 (83,3) | *ns* |
| Berufstätig | 8 (19,0) | 4 (66,7) | * |
| Unterstützung im Alltag benötigt | 8 (19,0) | – | *ns* |
| **Medikation pro Patient** (Median) | | | |
| Gesamtmedikation[b] | 9 [1-16] | 7 [4-11] | *ns* |
| Orale Antitumortherapeutika[b] | 2 [1-3] | 2 [1-2] | *ns* |
| Begleitmedikation | 8 [0-15] | 5 [2-10] | *ns* |
| OTC-Arzneimittel[a] | 1 [0-9] | 3 [0-7] | *ns* |
| **Lebenssituation** | | | |
| Mit Partner/Familie | 33 (78,6) | 6 (100,0) | *ns* |
| Alleinstehend | 8 (19,0) | – | |
| In Pflegeeinrichtung | 1 (2,4) | – | |
| **Tumorart** | | | |
| Melanom | 35 (83,3) | 5 (83,3) | *ns* |
| Kutanes T-Zell Lymphom | 5 (11,9) | 1 (16,7) | |
| Basalzellkarzinom | 2 (4,8) | – | |
| **Orale Antitumortherapeutika[d]** | | | |
| Dabrafenib/Trametinib | 24 (57,1) | 5 (83,3) | *ns* |
| Bexaroten | 4 (9,5) | 1 (16,7) | |
| Andere | 14 (33,3) | – | |
| **Behandlungscharakteristika** | | | |
| Zyklische Einnahme | 2 (4,8) | – | *ns* |
| Kurativ/adjuvant | 9 (21,4) | 3 (50,0) | *ns* |
| Off-label-Einsatz[e] | 5 (11,9) | – | *ns* |
| OAT Therapiebeginn (<7 Tage) | 30 (71,4) | 6 (100,0) | *ns* |

Adhärente Patienten mit „*Dosing Adherence*“ >80% und nicht-adhärente Patienten mit „*Dosing Adherence*“ ≤80%.

Abkürzungen: ECOG, Eastern Cooperative Oncology Group; NA, nicht zutreffend; OAT, orale Antitumortherapeutika; OTC, “over-the-counter” (freiverkäuflich). *Anmerkung:* Kategorische Variablen werden als Anzahl (%) der Patienten pro Gruppe angegeben, kontinuierliche Variablen als Mittelwert oder Median [Spannweite].

*p* < 0,05; ****p* < 0,001; ns, nicht signifikant (t-Test, Mann-Whitney-Test, $Chi^2$-Test oder Fishers’ Exact Test).

[a]Umfasst OTC-Arzneimittel und Nahrungs(ergänzungs)mittel.

[b]Umfasst Arzneimittel aller Applikationsarten (z.B. oral, parenteral oder topisch) und OTC-Arzneimittel, sowie Nahrungs(ergänzungs)mittel.

[c]Einer Patientin mit Melanom, die mit Dabrafenib/Trametinib behandelt wurde, wurde außerdem Exemestan bei Brustkrebs verordnet.

[d]Nur OAT, die für dermato-onkologische Indikationen verschrieben wurden, werden angeführt.

[e]Umfasst die palliative Therapie mit Temozolomid, Lenvatinib, Acitretin und die adjuvante Therapie mit Encorafenib/Binimetinib.
